# High-risk genotypes for type 1 diabetes are associated with the imbalance of gut microbiome and serum metabolites

**DOI:** 10.3389/fimmu.2022.1033393

**Published:** 2022-12-13

**Authors:** Tong Yue, Huiling Tan, Chaofan Wang, Ziyu Liu, Daizhi Yang, Yu Ding, Wen Xu, Jinhua Yan, Xueying Zheng, Jianping Weng, Sihui Luo

**Affiliations:** ^1^ Department of Endocrinology and Metabolism, The First Affiliated Hospital of USTC, Division of Life Sciences and Medicine, University of Science and Technology of China, Hefei, Anhui, China; ^2^ Department of Endocrinology and Metabolism, The Third Affiliated Hospital of Sun Yat-sen University, Guangzhou, Guangdong, China

**Keywords:** type 1 diabetes mellitus, human leukocyte antigen, gut microbiota, serum metabolites, serum lipids

## Abstract

**Background:**

The profile of gut microbiota, serum metabolites, and lipids of type 1 diabetes (T1D) patients with different human leukocyte antigen (HLA) genotypes remains unknown. We aimed to explore gut microbiota, serum metabolites, and lipids signatures in individuals with T1D typed by HLA genotypes.

**Methods:**

We did a cross-sectional study that included 73 T1D adult patients. Patients were categorized into two groups according to the HLA haplotypes they carried: those with any two of three susceptibility haplotypes (DR3, DR4, DR9) and without any of the protective haplotypes (DR8, DR11, DR12, DR15, DR16) were defined as high-risk HLA genotypes group (HR, n=30); those with just one or without susceptibility haplotypes as the non-high-risk HLA genotypes group (NHR, n=43). We characterized the gut microbiome profile with 16S rRNA gene amplicon sequencing and analyzed serum metabolites with liquid chromatography-mass spectrometry.

**Results:**

Study individuals were 32.5 (8.18) years old, and 60.3% were female. Compared to NHR, the gut microbiota of HR patients were characterized by elevated abundances of *Prevotella copri* and lowered abundances of *Parabacteroides distasonis*. Differential serum metabolites (hypoxanthine, inosine, and guanine) which increased in HR were involved in purine metabolism. Different lipids, phosphatidylcholines and phosphatidylethanolamines, decreased in HR group. Notably, *Parabacteroides distasonis* was negatively associated (p ≤ 0.01) with hypoxanthine involved in purine metabolic pathways.

**Conclusions:**

The present findings enabled a better understanding of the changes in gut microbiome and serum metabolome in T1D patients with HLA risk genotypes. Alterations of the gut microbiota and serum metabolites may provide some information for distinguishing T1D patients with different HLA risk genotypes.

## Introduction

Type 1 diabetes mellitus (T1D) is a disease driven by genetic and environmental factors (1). Human leukocyte antigen (HLA) allele combinations are the most significant genetic risk factors for the development of T1D (2). The HLA epitopes have been shown to be protective (like HLA-DR14, DR15) or detrimental (like HLA-DR3, DR4, DR9) with susceptibility for T1D (1, 3). Moreover, HLA-DR3 (4), DR4 (5), and DR9 (6) are also observed to be associated with cardiometabolic risk, an important cause of death for patients with T1D.

The gut microbiome is a vital environmental factor of T1D that has been increasingly studied in recent years (7). The imbalance of the gut microbiota may modify intestinal immunity as well as alter intestinal permeability (8), which mediate the consequent imbalance of metabolites (9), islet autoimmunity (10, 11), and the development of T1D (12). Serum metabolites and lipids are also environmental factors involved in T1D pathogenesis (1). Multiple serum metabolites and lipid molecules have been found to vary between patients with T1D and healthy populations (13). Also, a recent study suggested that gut-related metabolites are associated with autoimmunity and pathogenesis of latent autoimmune diabetes in adults, suggesting that the interaction between gut microbiota and diabetes could be mediated by certain metabolites (14). Furthermore, the gut microbiota and serum metabolites of T1D patients are also associated with the progression of microvascular (15, 16) and macrovascular complications (16, 17).

Interestingly, HLA can alter gut microbiota (18, 19). Animal and human studies confirmed that HLA-DR can influence the development of autoimmune diseases by shaping the microbiome (20). HLA-DR3 and DR4 are common HLA risk haplotypes for autoimmune diseases, which can change the diversity of gut microbiome in the development of multiple sclerosis (21), autoimmune hepatitis (22), and rheumatoid arthritis (23). However, studies about the impact of HLA on the gut microbiome in T1D animal models and patients are limited. In one study, specific major histocompatibility complex (MHC) alleles prevent T1D in NOD mice by shaping intestinal microbes (24). However, only two studies in humans reported that the HLA risk for developing T1D is associated with the gut microbiome changes (12, 25). Also, to date, the effect of T1D related-HLA on serum metabolites and lipids has not been reported in previous studies or investigated in relation to gut microbiota. Therefore, it remains poorly understood how the HLA, gut microbiome, serum metabolites, and lipids interact within the host so as to result in the development of or protection from T1D.

This study used an integrative multi-omics analysis to explore the intestine microbiome and serum metabolites profile in T1D patients with different HLA genotypes. We further discussed whether HLA-associated perturbation of the microbiome and metabolite profile might influence the T1D development and cardiovascular risk in T1D patients.

## Results

### Clinical characteristics of the T1D patients

In this cross-sectional study, we included a total of 73 patients with T1D. The recruitment details were available in the methods sections. We categorized these patients into two groups according to the HLA haplotypes they carried: those with any two of three susceptibility haplotypes (DR3, DR4, DR9) and without any of the protective haplotypes (DR8, DR11, DR12, DR15, DR16) were defined as high-risk HLA genotypes group (HR, n=30, 70.0% female); those with just one or without susceptibility haplotypes as the non-high-risk HLA genotypes group (NHR, n=43, 53.5% female). The average age of the patients in the HR group and the NHR group was 31.7 (6.76) years and 33.2 (9.07) years, respectively. No significant differences in most characteristics such as age and sex were observed between the HR and the NHR groups. Moreover, the prevalence of glutamic acid decarboxylase autoantibody (GADA) positivity was higher in the HR group (*p*-value: 0.025). More clinical characteristics information is shown in **Table 1**.

**Table 1:** Clinical characteristics of the T1D patients.

Clinical characteristics	NHR (N=43)	HR (N=30)	*p*-value
Age (year)	33.2 (9.07)	31.7 (6.76)	0.422
Male	20 (46.5%)	9 (30.0%)	0.240
BMI (kg/m^2^)	21.0 (2.08)	21.7 (1.84)	0.151
TC (mmol/l)	4.98 (0.89)	4.57 (0.71)	0.033
TG (mmol/l)	0.66 [0.53;0.89]	0.68 [0.54;0.92]	0.801
HDLC (mmol/l)	1.54 (0.32)	1.52 (0.28)	0.745
LDLC (mmol/l)	3.00 (0.80)	2.69 (0.54)	0.052
HbA1c (%)	7.00 [6.15;7.85]	7.15 [6.32;7.50]	0.801
Fasting C peptide (nmol/l)	0.02 [0.02;0.03]	0.02 [0.02;0.02]	0.058
Islet Autoantibodies (+)	23 (53.5%)	23 (76.7%)	0.076
GADA (+)	16 (37.2%)	20 (66.7%)	0.025
ZnT8A (+)	6 (14.0%)	8 (26.7%)	0.291
IA2A (+)	12 (27.9%)	10 (33.3%)	0.812
Age of onset (year)	21.4 (9.09)	18.1 (7.95)	0.111
Diabetes duration (year)	10.7 [8.25;14.1]	12.2 [9.62;16.4]	0.173
Insulin dosage (u/kg)	0.70 [0.54;0.81]	0.67 [0.54;0.79]	0.642
Energy Intake (kcal/day)	1185 [1020;1687]	1196 [825;1509]	0.346
Fat Intake (g/day)	42.1 [30.8;57.0]	35.3 [27.0;49.4]	0.148
Protein Intake (g/day)	66.9 [52.0;80.8]	60.2 [48.7;80.9]	0.523
Exercise (min/week)	88.2 [29.4;135]	44.1 [14.7;197]	0.454
Alcohol (No)	27 (62.8%)	19 (63.3%)	1.000
Smoking (No)	38 (88.4%)	25 (83.3%)	0.731

Continuous variables in a normal distribution are shown with mean (SD), while the median [interquartile range] is used to show the non-normally distributed data. Total numbers, n (percentage), are used to represent categorical variables; TG, Triglyceride; TC, total cholesterol; LDLC, low-density lipoprotein cholesterol; HDLC, high-density lipoprotein cholesterol; HbA1c, glycosylated haemoglobin A1c; GADA, glutamic acid decarboxylase autoantibodies; ZnT8A, zinc transporter 8 antibody; IA‐2A, anti‐protein tyrosine phosphatase like protein.

### Microbiome community profiling of HR and NHR

**Figure 1:**
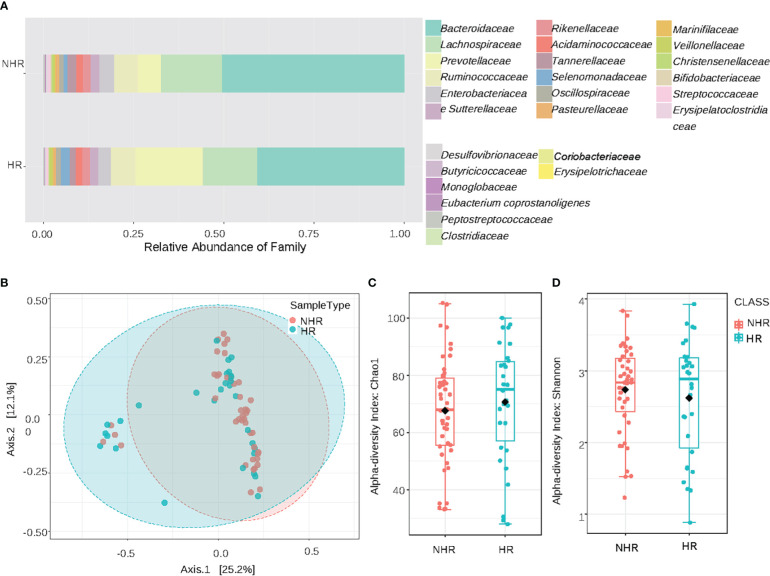
Results of diversity and taxonomy. **(A)** At the family level,the stacked bar plots; **(B)** Result of β-diversity visualized using principal coordinate analysis based on Bray-Curtis Index (Permutational MANOVA test, F= 1.8078, R2= 0.02483, p<0.066); **(C, D)** The plots of α-diversity: **(C)** The chao1 -diversity boxplots (T-test, p= 0.51802). **(D)** The shannon index boxplots (T-test, p=0.51787).

The overview of the relative abundances at the family levels is displayed in **Figure 1A**. Other taxonomic levels results are shown in the Supplementary Materials **Supplementary Figures 1A–C, E, F**. The β-diversity plots are demonstrated in **Figure 1B** (Permutational multivariate analysis of variance test, F= 1.8078, R2 = 0.02483, *p<*0.066). For the α-diversity analysis, the Chao1 index (T-test, *p=* 0.51802) and the Shannon index (T-test, *p=* 0.51787) showed no statistical differences between the NHR and HR groups (**Figures 1C, D**). Results of the ACE index (T-test, *p=* 0.99438), Simpson index (T-test, *p=* 0.30753) and Fisher index (T-test, *p=* 0.50573) are shown in the supplementary materials **Supplementary Figure 1D**. From the pattern, the microbiota profiles between HR and NHR groups appeared to be different, though the differences were not statistically significant.

**Figure 2:**
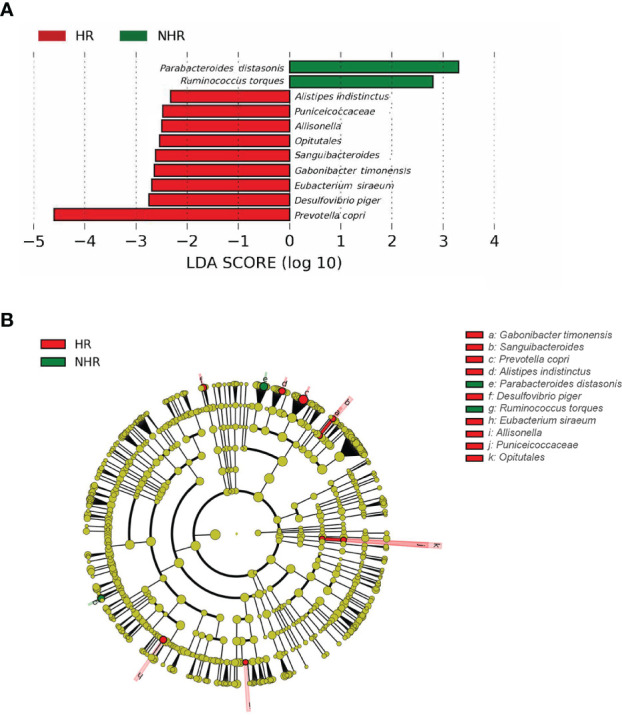
Results of different taxa (screened by P value <0.05) between two groups by LEfSe analysis. **(A)** Histogram of LDA value distribution. **(B)** Cladogram plots depicting the microbial taxa that differed significantly.

We further investigated the differential microbiota composition between the two groups by the linear discriminant analysis effect size analysis (LEfSe). The threshold of the logarithmic LDA score for discriminative features was 2. The histogram of LDA value distribution and the cladogram of different taxa is demonstrated in **Figures 2A, B**. *Prevotella copri* (FDA=4.56519; *p=*0.0281) and *Parabacteroides distasonis* (FDA=3.24702; *p=*0.04845) were of the most significantly difference in HR and NHR group, respectively. The different relative abundance of *Parabacteroides distasonis* (T−test, p = 0.039) in the HR and NHR groups was shown in **Supplementary Figure 2A**, and the different relative abundance of *Prevotella copri* (Wilcoxon, p = 0.029) was shown in **Supplementary Figure 2B**. Besides, some microbes with FDA more than 2 to less than 3 were observed. At the species level, the *Ruminococcus torques* abundance was lower in the HR group, but the abundance of *Gabonibacter timonenis*, *Alistipes indistinctus*, and *Desulfovibrio piger* were higher. At the order level, *Opitutales* was elevated in the HR group, consistent with the elevated abundance of *Puniceicoccaceae*, which belonged to *Opitutales* (**Figure 2A**).

### Metabolites and lipids profiles of HR and NHR

**Figure 3:**
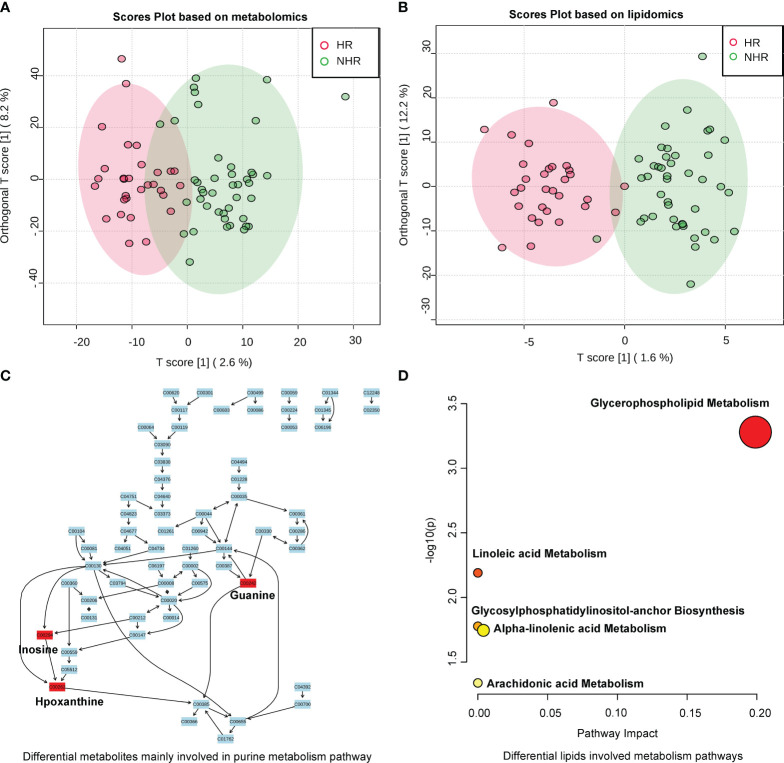
Results of metabolites profiles analysis between two groups. **(A)** OPLS-DA analysis displaying a discriminative trend of metabolite composition and **(B)** lipid composition between two groups. **(C)** Differential metabolites (screened by combining P value (<0.05) and fold­ change (>1.2)) mainly involved in purine metabolism (Hypoxanthine;Inosine;Guanine). **(D)** Differential lipid metabolite were phosphatidylcholines and phosphatidylethanolamines, which involved in glycerophospholipid, linoleic acid, alpha-linolenic acid, arachidonic acid metabolism, and glycosylphosphatidylinositol-anchor biosynthesis.

Different compositions of serum metabolites and lipids were observed between the two groups according to latent structure discriminant analysis (OPLS-DA) (**Figures 3A, B**). Furthermore, 155 differential metabolites and 22 lipids were screened by combining fold-change (>1.2) and p-value (<0.05). The results are shown in volcano plots in supplemental materials **Supplementary Figure 3A, B**. Compared with the NHR group, 3a,7a-dihydroxycholanoic acid, C23H45P3, C16H39N4O3P, diisodecyl phthalate, C26H46O4P2 were differential metabolites that decreased in the HR group, while C29H62ClN4O5P, hypoxanthine, inosine, and guanine increased. As for differential lipids, most of them belonged to glycerophospholipids. phosphatidylcholine (35:1), phosphatidylcholine (33:1), phosphatidylethanolamine (36:5), and phosphatidylcholine (37:4) decreased in the HR group, and phosphatidylethanolamine (38:3), lysodimethylphosphatidylethanolamine (19:4), phosphatidylethanolamine (40:6) increased, compared with the NHR group.

We then annotated the differential metabolites and lipids in Human Metabolome Database to perform pathway analysis. Among the 155 differential metabolites and 22 lipids, 62 differential metabolites and 16 lipids were annotated. The heat map in supplemental materials **Supplementary Figures 4A, D** showed the differential levels of these annotated metabolites and lipids between the HR and the NHR groups. In the pathway analysis of differential metabolites, the purine metabolism pathway was annotated (p = 0.031421) in the Kyoto Encyclopaedia of Genes and Genomes database, and three differential metabolites (hypoxanthine, inosine, and guanine) were involved. **Figure 3C** demonstrated the purine metabolism pathway, showing differential metabolites (hypoxanthine, inosine, and guanine) elevated in the HR group. In the pathway analysis of differential lipids, most of the differential lipids were phosphatidylcholine and phosphatidylethanolamine, which are involved in the metabolism of glycerophospholipid, linoleic acid, alpha-linolenic acid, and arachidonic acid; and glycosylphosphatidylinositol-anchor biosynthesis **Figure 3D**.

### Correlation between HLA-risk differential serum metabolites and gut microbes

**Figure 4:**
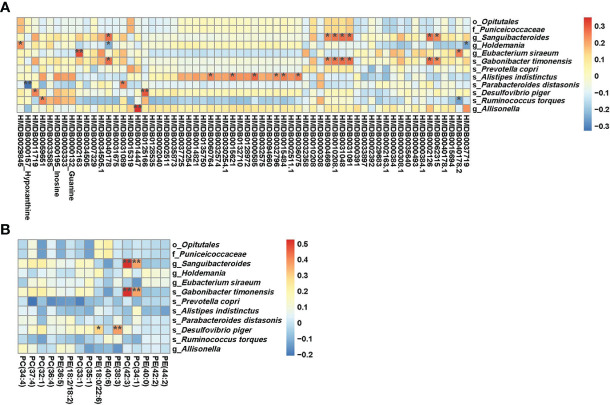
**(A)** Results of combined analysis of microbiome and metabolome between two groups; **(B)** Results of combined analysis of microbiome and lipidome between two groups. **p<=0.01,*p<=0.05.

Association analyses of HLA-risk differential gut microbiota and differential serum metabolites demonstrated that *Parabacteroides distasonis* was negatively associated (r=-0.32701, p ≤ 0.01) with hypoxanthine involved in the purine metabolic pathways, which was presented in the **Figure 4A**. As to *Prevotella copri*, another differential microbe with LDA>3, there was no relationship between *Prevotella copri* and differential metabolites. Moreover, the level of microbes with FDA more than 2 to less than 3 were associated with differential metabolites. The *Holdemania* was negatively associated with butylhydroquinone (HMDB0040178). The *Desulfovibrio piger* was positively associated with homovanillic acid sulfate (HMDB0011719), and the *Allisonella* was positively associated with tranexamic acid (HMDB0014447).

The association analyses of differential gut microbiota and differential lipids were presented in **Figure 4B**. Some kinds of phosphatidylcholine (phosphatidylcholine (34:1), phosphatidylcholine (42:3)) showed a positive association with Sanguibacteroides and Gabonibacter timonensis. The *Desulfovibrio piger* was positively associated with phosphatidylethanolamines. However, there was no relationship between *Parabacteroides distasonis* and differential lipids. *Prevotella copri* also showed no relationship with differential lipids.

## Discussion

Our results demonstrated that HLA appears to perturb the gut microbiome and serum metabolite profile among patients with T1D. To our knowledge, this is the first time to explore the multi-omics profile, including the gut microbiome, serum metabolome, and lipidome, in T1D subjects with different genetic risks. Most previous studies compared the different microbiome profiles between T1D participants and healthy controls both at high T1D-HLA genetic risk (26–30). To date, the effect of T1D-HLA genetic risk on serum metabolites and lipids has not been reported, which may link HLA genotype risks and relative microbiota changes. Our results provided insight into understanding the interaction of HLA genetic risk and environmental risks in T1D.

In our study, the profile of gut microbiome, serum metabolite, and lipidome between patients with different T1D-HLA genetic risks (DR3/4/9) are different. The influence of T1D-related genes on gut bacterial composition has been explored in previous animal studies (24, 31). However, only a few studies reported that HLA genotypes with a higher risk for developing T1D are associated with human gut microbiome changes (12, 25). They found that T1D patients with both DR3/DR4 haplotypes had the lowest responses to intestinal commensal bacteria such as *R. faecis* (12). In addition, *Saccharimonadaceae* is elevated in subjects with high-risk HLA haplotypes (DR3/DR4 only), *Romboutsia* and *Intestinibacter* are elevated in people with protective haplotypes (25). The mechanism under the HLA effect on regulating gut microbiota in T1D may involve innate and adaptive immune responses. HLA molecules are antigen presentation molecules that can present peptides to CD4+ T cells (32), and the HLA polymorphism can recognize unique bacterial antigens that caused the depletion of these gut taxa and the changing gut bacterial composition. As to the effect of T1D-HLA genetic risk on serum metabolites and lipids, the related studies were limited. However, we found a negative association between *Parabacteroides distasonis* and hypoxanthine in our study, suggesting that the HLA may indirectly influence the profile of serum metabolites by regulating the gut microbiota.

The differential gut microbiota, serum metabolites, and lipids between the HR and NHR groups are found to be related to the development of T1D or other human autoimmune diseases in previous studies. In our study, we found that HR patients were characterized by enriched *Prevotella copri, Desulfovibrio piger, Eubacterium siraeum, Sanguibacteroides, Gabonibacter timonensis, Puniceicoccaceae, Opitutales, Allisonella, Alistipes indistinctus*, and *Holdemania*. NHR patients were characterized by enriched *Parabacteroides distasonis* and *Ruminococcus torques*. Microbiota populations of *Prevotella, Parabacteroides distasonis, and Ruminococcaceae* are related to insulin signalling pathway and carbohydrate metabolism, which are also enriched in gestational diabetes mellitus populations (33). Consistent with our results, the abundance of *Prevotella copri* increases in many human autoimmune diseases, such as rheumatoid arthritis (34), ankylosing spondylitis (35), and T1D (36). Studies have indicated that *Prevotella copri* can aggravate the branched-chain amino acids synthesis as well as induce in insulin resistance (37). However, another study results demonstrated that *Prevotella copri* is an indicator of good postprandial glucose metabolism (38), and the glucose metabolism improvement induced by plant diet is linked to the increased *Prevotella copri* (39, 40). Nevertheless, Eleftherios P Diamandis held that *Prevotella copri* proliferate in a specific dietary and lifestyle milieu are epiphenomena for a lifestyle (41). Enrichment of *Prevotella copri* is predictive for Western individuals responding favorably to a high-fiber, barley-kernel-based diet (42). These studies (41, 42) indicated the influence of diet on the abundance and contrasting effect of *Prevotella copri*. Further mechanism study of *Prevotella copri* in relation to T1D is needed.

In addition, *Desulfovibrio piger* is another microbe enriched in the HR group, which was reported to flourish in aged, immunocompromised, and glucose regulation impaired obese mice (43). Expansion of *Desulfovibrio* was key features of humans with metabolic syndrome. In addition, in mice fed the Methylococcus-based western diet (a diet can improve glucose regulation), researchers found the diet-induced glucose changes were consistent with the substantial reduction of *Desulfovibrio* abundance (44).

Another differential microbiota *Parabacteroides distasonis* enriched in the NHR group, is also related to the development of T1D. Accumulating evidence suggests *Parabacteroides distasonis* is a potential probiotic that can exert protective effects against diabetes, multiple sclerosis, and cardiovascular disease (CVD) (45). Wang et al. reported that *Parabacteroides distasonis* can alleviate metabolic dysfunctions and obesity. They treated obese mice with live *Parabacteroides distasonis*, resulting in less food intake and ameliorating glucose homeostasis (46). However, several studies have suggested that *Parabacteroides distasonis* 33B can mimic the human insulin B:9–23 peptide and may stimulate T1D onset (47). Different *Parabacteroides distasonis* strains and host genetic background may be the potential decisive factors of those contrasting findings.

Differential serum metabolites detected in this study were concentrated around the purine metabolism pathway, and the association between purine metabolism and diabetes, including T1D (48), T2D (49) as well as gestational diabetes mellitus (50), has been investigated in previous studies. Consistent with our results, elevated serum hypoxanthine and uridine levels had been found in T1D patients (51). In addition, one previous study showed that uric acid, xanthine, inosine, and adenosine levels are elevated in T2D patients with diabetic nephropathy, which indicates those purine metabolites may be helpful in evaluating the development of T2D (52).

As to the differential lipid metabolites, the level of most phosphatidylcholines and phosphatidylethanolamines was lower in the HR group. The dysregulation of lipid metabolism can precede T1D in earlier metabolomics studies (53). For example, compared to children who did not progress to T1D, the phosphatidylcholine in children who progressed to T1D is downregulated (54). Another study observed the association between phosphatidylcholine and reversion of islet autoimmunity, which implied the prevention of T1D progression (55). In young age-at-onset T1D children, the decreased glycerophospholipids in cord blood also predicts a high risk for T1D progression and islet autoimmunity (56, 57).

Notably, in our study, we also speculated that HLA-associated perturbation of the microbiome and metabolite profile might increase the cardiovascular risk in T1D patients. As discussed before, *Prevotella copri* (58) and *Parabacteroides distasonis* (59) are related to the pathogenesis of CVD. The purine metabolites are associated with CVD risk and mortality in T1D patients (60). The differential lipid metabolite phosphatidylcholine and unsaturated fat metabolism were also related to the CVD mortality and total mortality in T2D patients (61). Though the people included in this survey self-reported no history of CVD, we will continue to trace the CVD status of those patients to verify the conjecture.

The limitations of this study are as follows. First, the sample size is relatively small. Second, the overestimation of the causalities in microbiota studies is common, and we should look at this study’s results with caution. Third, the gut microbiota dynamics can’t be discovered in this study because it’s a cross-sectional study. One of the strengths of this study is the well-characterized subjects. Moreover, the measurement of serum and lipid metabolites is targeted and quantitative. Compared to previous studies, this study first explores the multi-omics profile in T1D patients with different HLA gene risks.

## Conclusion

In conclusion, we depicted the multi-omics profile, including the gut microbiota, serum metabolites, and lipids in T1D subjects with different HLA genotypes. High-risk T1D-related HLA genotypes might perturb the profile of microbiome and metabolite in T1D patients. We further speculated that HLA-associated perturbation of the microbiome and metabolite profile might increase the cardiovascular risk in T1D patients. Although the specific mechanism of HLA on the microbiome and metabolome is still unclear, our findings will provide some information to help better understand the association between HLA and microbiome, and provide some information for distinguishing T1D patients with different HLA risk genotypes.

## Methods

### Subject recruitment and sample collection

There were 73 individuals with T1D admitted to the Department of Endocrinology of the Third Affiliated Hospital of Sun Yat-sen University recruited in this cross-sectional study from 2019 to 2020 (62). The criteria of the American Diabetes Association were used to make the diagnosis of T1D (63). Chronic gastrointestinal disorders, chronic or acute inflammatory and infectious disorders, usage of antibiotic medicine, probiotics, and corticosteroids within three months after enrolment, pregnancy, and breastfeeding were all exclusion factors. All participants were given diabetes instructions and were required to follow a diabetes diet. High-resolution HLA DRB1-DQA1-DQB1 haplotypes sequencing was performed by sequence specific oligonucleotide, which would be used to perform the HLA genotyping. In Asian populations, DRB1*0301-DQA1*0501-DQB1*0201, DRB1*0405-DQA1*0301, DRB1*0901-DQB1*0303 were the common susceptible haplotypes for the development of T1D (64, 65). DRB1*0803-DQA1*0103-DQB1*0601, DRB1*1101-DQB1*0301, DRB1*1202-DQA1*0601-DQB1*0301, DRB1*1501-DQA1*0102-DQB1*0602 (64, 66), and DRB1*16 (67) were the protective haplotypes for T1D. Based on the evidence from studies mentioned above, we classified HLA DRB1-DQA1-DQB1 haplotypes into three categories, susceptibility haplotype (DR3, DR4, DR9 haplotype), protective haplotype (DR8, DR11, DR12, DR15, DR16 haplotype) and others (Supplementary Materials ST. 1). Next, we defined a criterion to group patients with high and non-high risk HLA genotypes based on haplotype properties (Supplementary Materials ST. 2) (64, 68). In detail, patients with any two of three risk haplotypes (DR3, DR4, DR9 haplotype) and without any of the protective haplotypes (DR8, DR11, DR12, DR15, DR16 haplotype) were defined as high-risk HLA genotypes group (HR); patients with just one or without risk haplotypes as the non-high-risk HLA genotypes group (NHR). In result, patients were categorized into HR (n=30) and NHR (n=43) groups.

Personal and medical history were obtained by interview and electronic patient records (**Table 1**). In total, 73 fecal samples (30 HR patients and 43 NHR patients) were collected and stored in a sterilized tube, and transformed to the laboratory to keep at -80°C for further analysis. Blood samples for metabolites analysis were also obtained from all participants and stored at -80°C till processing.

### Microbial 16S rRNA gene sequence analysis

Microbial 16S rRNA gene sequencing was carried out on the Illumina platform with the paired-end sequencing strategy. Briefly, we followed the instructions of MagPure Stool DNA KF kit B (Magen, China) and extracted DNA from fecal samples first. Then we did PCR with primers 806R and 341F to amplify the bacterial 16S rRNA gene’s V3-V4 regions. Next, we purified V3-V4 amplicons with the AmpureXP beads and eluted amplicons in an Elution buffer. Finally, purified V3-V4 amplicons received the paired-end sequencing on an Illumina platform.

MOTHUR (v1.31.2) was used to splice and process the raw 16S rRNA gene amplicons to get high-quality sequencing (69). Then high-quality gene amplicons were analyzed in QIIME package for gene amplicon sequence variants (ASVs) classification (70). The QIIME v1.8.0 with Greengenes database v201305 was used as the reference database for classifying ASVs. Based on ASVs annotation, taxonomic profiles were created at different levels (the level of phylum, class, order, family, genus).

Various alpha diversity indexes such as the Shannon diversity index were used to assess gut microbial community richness variations between the HR and NHR groups. The global microbiota composition and structure differences (beta diversity) of the two groups based on ASV abundance were compared using principal coordinate analysis with the Bray-Curtis Index. Those analyses were performed on the MicrobiomeAnalyst website (71, 72). Furthermore, LEfSe (73) was carried out on the Galaxy website (74) to determine the significantly different taxa between HR and NHR groups.

### Serum metabolomics and lipidomics analysis

Untargeted liquid chromatography-tandem mass spectrometry analysis was used to detect serum small molecule metabolites. High-resolution mass spectrometer Q Exactive (Thermo Fisher Scientific, USA) was used in negative and positive ion modes to increase the coverage of lipid detection.

Metabolome analysis was performed on the MetaboAnalyst website (75, 76). For the detected small molecule metabolites, orthogonal projection to OPLS-DA was used to examine the overall plastic metabolites distribution as well as to detect differential metabolites between the HR and NHR groups. Combining fold-change (>1.2) and *p*-value (<0.05) was used to screen out the differential serum metabolites. Then the signal transduction pathways and biochemical metabolic pathways of differential metabolites were annotated in the Kyoto Encyclopaedia of Genes and Genomes database.

### Combined analysis of microbiome and metabolome

A combined analysis was constructed based on Pearson correlation analysis to investigate the complex relationship between microbiome and metabolome. Briefly, differential metabolites and microbes detected before were selected to calculate the correlation coefficient and statistical significance using the R package psych 2.1.9.

### Statistical analysis

Statistical analyses were done on R version 4.1.1 (http://www.r-project.org/). The clinical characteristics data analysis was performed with the R package compareGroup 4.5.1, Shapiro-Wilks test was performed first to decide the continuous variable was normal or non-normal-distributed. Then the continuous variables were compared between groups using t-test or analysis of variance. Categorical data were compared by exact Fisher test or Chi-square test. For microbiome and metabolome data analysis, specific matched statistics methods and websites were used, as mentioned above. *P* values less than 0.05 were considered significant.

### Ethics approval

The Ethics Committee of The Third Affiliated Hospital of Sun Yat-sen University approved this study. Our study was conducted based on the Declaration of Helsinki. All subjects provided written informed consent.

## Data availability statement

The datasets presented in this study can be found in online repositories. The names of the repository/repositories and accession number(s) can be found below: https://www.ncbi.nlm.nih.gov/, PRJNA766410.

## Ethics statement

The studies involving human participants were reviewed and approved by The Ethics Committee of The Third Affiliated Hospital of Sun Yat-sen University. The patients/participants provided their written informed consent to participate in this study.

## Author contributions

JPW, XYZ, and SHL contributed to the conception of the study. ZYL and DZY contributed to data acquisition. TY, CFW, JHY and WX contributed to the analysis. HLT and TY drafted the manuscript. YD and CFW revised the manuscript. All authors contributed to the article and approved the submitted version.
