# Supplementary material for: High-risk genotypes for type 1 diabetes are associated with the imbalance of gut microbiome and serum metabolites
Source: Front Immunol. 2022 Dec 13;13:1033393. doi: 10.3389/fimmu.2022.1033393 (PMC9794034; doi:10.3389/fimmu.2022.1033393)
Supplement: Supplementary file 1 [file DataSheet_1.pdf]

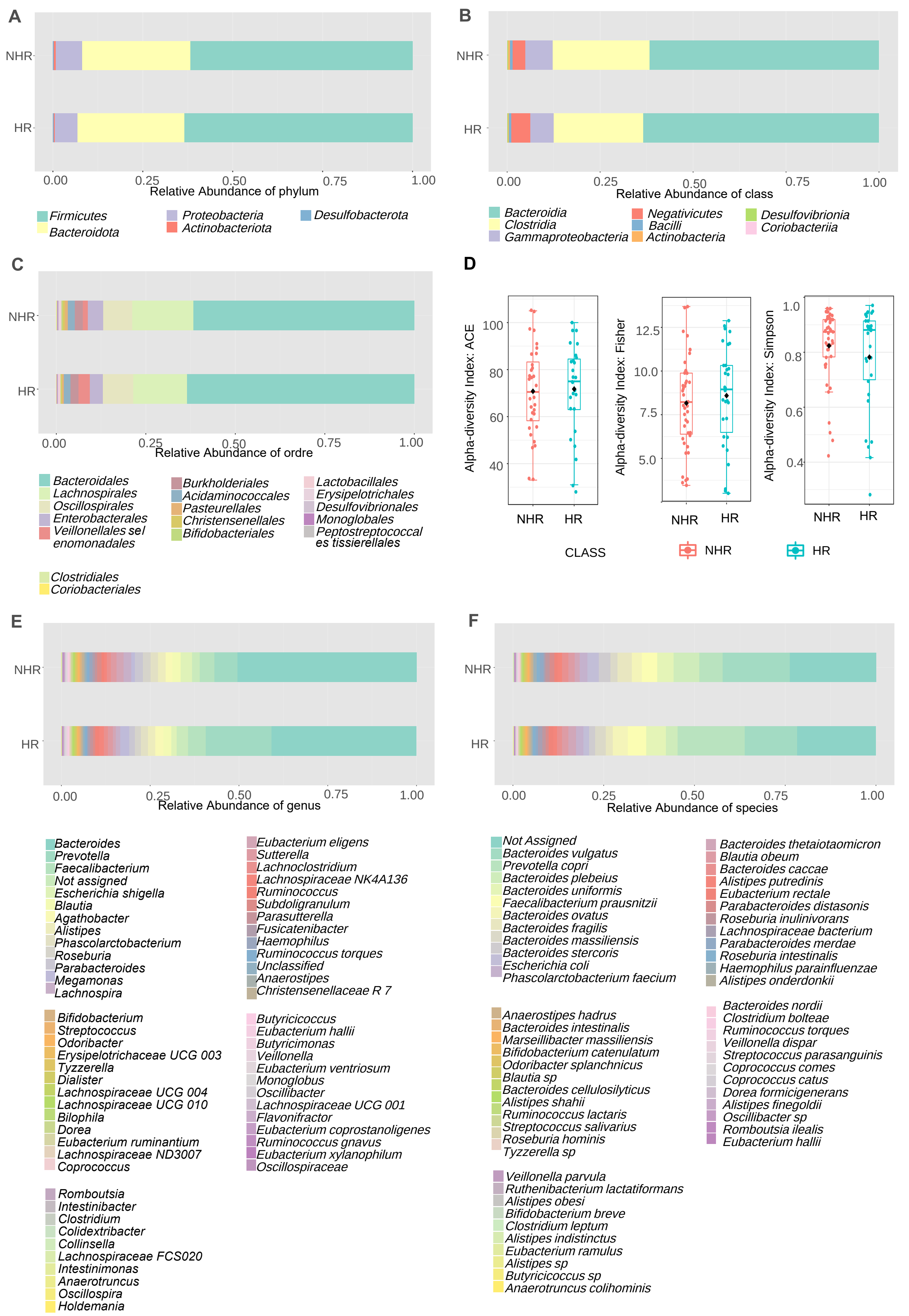
A
NHR
HR
0.00
0.25
0.50
0.75
1.00
Relative Abundance of phylum
Firmicutes
Bacteroidota
Proteobacteria
Actinobacteriota
Desulfobacterota
B
NHR
HR
0.00
0.25
0.50
0.75
1.00
Relative Abundance of class
Bacteroidia
Clostridia
Gammaproteobacteria
Negativicutes
Bacilli
Actinobacteria
Desulfovibrionia
Coriobacteriia
C
NHR
HR
0.00
0.25
0.50
0.75
1.00
Relative Abundance of ordre
Bacteroidales
Lachnospirales
Oscillospirales
Enterobacterales
Veillonellales sel enomonadales
Burkholderiales
Acidaminococcales
Pasteurellales
Christensenellales
Bifidobacteriales
Lactobacillales
Erysipelotrichales
Desulfovibrionales
Monoglobales
Peptostreptococcal es tissierellales
Clostridiales
Coriobacteriales
D
Alpha-diversity Index: ACE
100
80
60
40
Alpha-diversity Index: Fisher
12.5
10.0
7.5
5.0
Alpha-diversity Index: Simpson
1.0
0.8
0.6
0.4
NHR
HR
CLASS
NHR
HR
E
NHR
HR
0.00
0.25
0.50
0.75
1.00
Relative Abundance of genus
Bacteroides
Prevotella
Faecalibacterium
Not assigned
Escherichia shigella
Blautia
Agathobacter
Alistipes
Phascolarctobacterium
Roseburia
Parabacteroides
Megamonas
Lachnospira
Eubacterium eligens
Sutterella
Lachnoclostridium
Lachnospiraceae NK4A136
Ruminococcus
Subdoligranulum
Parasutterella
Fusicatenibacter
Haemophilus
Ruminococcus torques
Unclassified
Anaerostipes
Christensenellaceae R 7
Bifidobacterium
Streptococcus
Odoribacter
Erysipelotrichaceae UCG 003
Tyzzerella
Dialister
Lachnospiraceae UCG 004
Lachnospiraceae UCG 010
Bilophila
Dorea
Eubacterium ruminantium
Lachnospiraceae ND3007
Coprococcus
Butyricicoccus
Eubacterium hallii
Butyricimonas
Veillonella
Eubacterium ventriosum
Monoglobus
Oscillibacter
Lachnospiraceae UCG 001
Flavonifractor
Eubacterium coprostanoligenes
Ruminococcus gnavus
Eubacterium xylanophilum
Oscillospiraceae
Romboutsia
Intestinibacter
Clostridium
Colidextribacter
Collinsella
Lachnospiraceae FCS020
Intestinimonas
Anaerotruncus
Oscillospira
Holdemania
F
NHR
HR
0.00
0.25
0.50
0.75
1.00
Relative Abundance of species
Not Assigned
Bacteroides vulgatus
Prevotella copri
Bacteroides plebeius
Bacteroides uniformis
Faecalibacterium prausnitzii
Bacteroides ovatus
Bacteroides fragilis
Bacteroides massiliensis
Bacteroides stercoris
Escherichia coli
Phascolarctobacterium faecium
Bacteroides thetaiotaomicron
Blautia obeum
Bacteroides caccae
Alistipes putredinis
Eubacterium rectale
Parabacteroides distasonis
Roseburia inulinivorans
Lachnospiraceae bacterium
Parabacteroides merdae
Roseburia intestinalis
Haemophilus parainfluenzae
Alistipes onderdonkii
Anaerostipes hadrus
Bacteroides intestinalis
Marseillibacter massiliensis
Bifidobacterium catenulatum
Odoribacter splanchnicus
Blautia sp
Bacteroides cellulosilyticus
Alistipes shahii
Ruminococcus lactaris
Streptococcus salivarius
Roseburia hominis
Tyzzerella sp
Bacteroides nordii
Clostridium bolteae
Ruminococcus torques
Veillonella dispar
Streptococcus parasanguinis
Coprococcus comes
Coprococcus catus
Dorea formicigenerans
Alistipes finegoldii
Oscillibacter sp
Romboutsia ilealis
Eubacterium hallii
Veillonella parvula
Ruthenibacterium lactatiformans
Alistipes obesi
Bifidobacterium breve
Clostridium leptum
Alistipes indistinctus
Eubacterium ramulus
Alistipes sp
Butyricicoccus sp
Anaerotruncus colihominis

SF. 1. Supplemental results of diversity and taxonomy. (A-C and E-F) The stacked bar plots at phylum (A), class (B), order (C), genus (E), species (F) level. (D) The boxplots of α-diversity of ACE, Simpson and Fisher index.

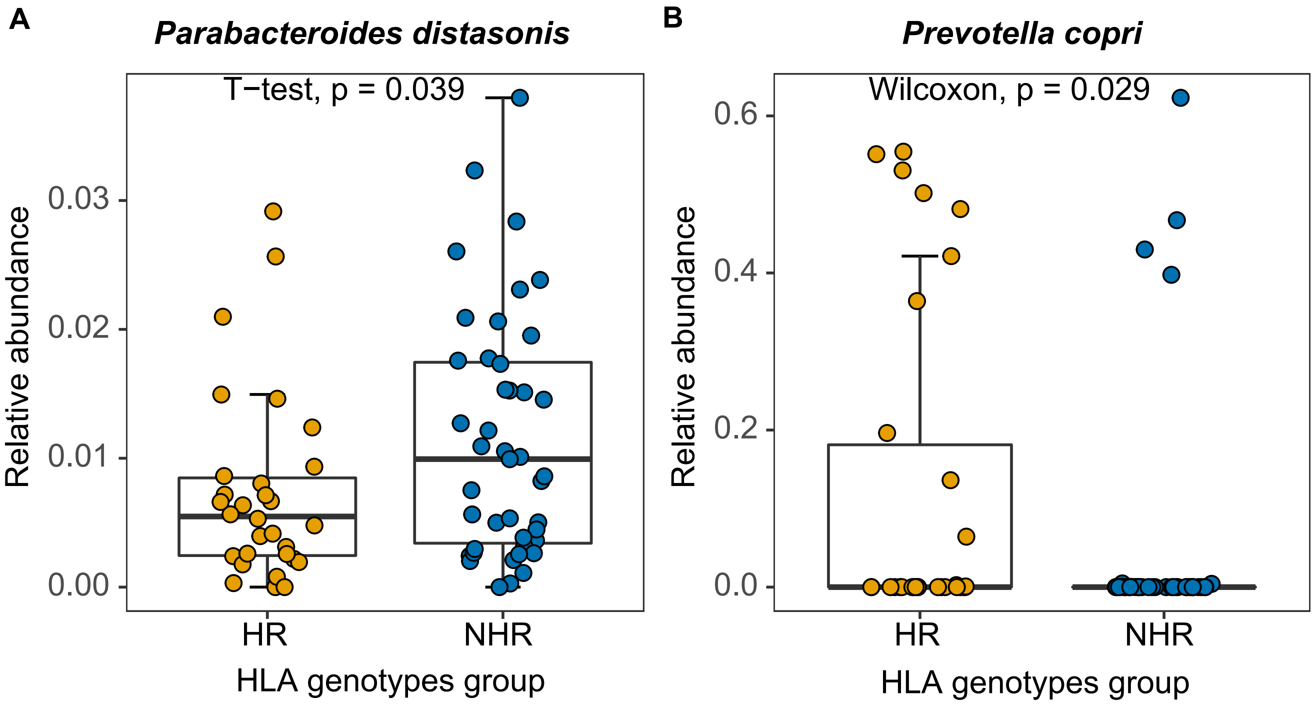

SF. 2. (A and B) Relative abundance of *Parabacteroides distasonis* (A) and *Prevotella copri* (B) in HR and NHR groups.

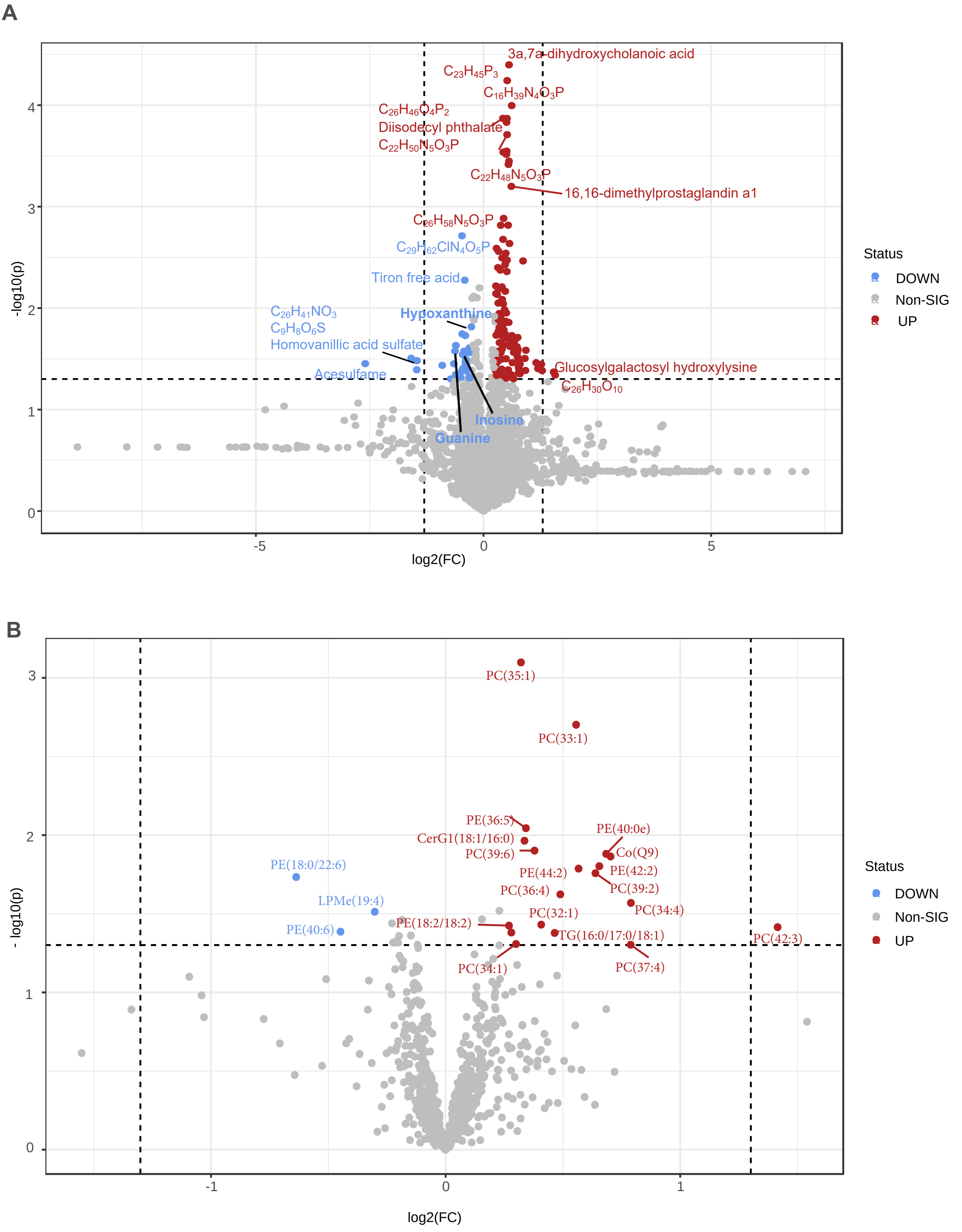

SF. 3. Volcano plots showing the differential metabolites (A) and lipids (B) screened by combining P value (<0.05) and fold-change (>1.2). The significance was determined by using t-tests.

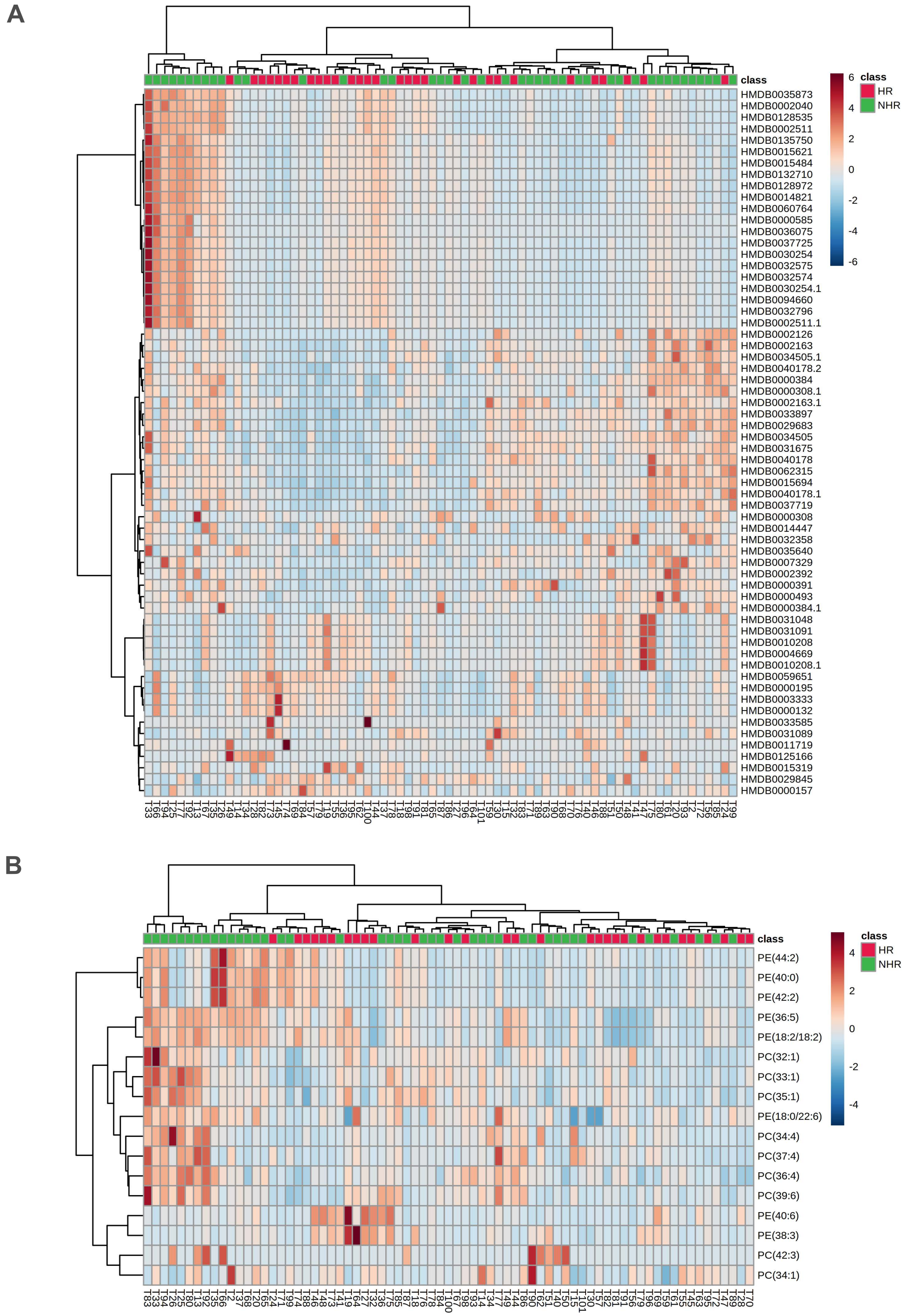

SF. 4. (A and B) Heat map showing the Human Metabolome Database annotated metabolites (A) and lipids (B) that different between the HR and NHR groups.

ST. 1. Classification of HLA DRB1-DQA1-DQB1 haplotypes based on their risk for T1D.

HLA DRB1-DQA1-DQB1 haplotype	Risk
(DR3) DRB1*030101-DQA1*050101-DQB1*020101	susceptibility
(DR4) DRB1*040501-DQA1*030301-DQB1*040101	susceptibility
(DR4) DRB1*040501-DQA1*030101-DQB1*030201	susceptibility
(DR4) DRB1*040501-DQA1*030301-DQB1*030201	susceptibility
(DR9) DRB1*090102-DQA1*030201-DQB1*030302	susceptibility
(DR8) DRB1*080302-DQA1*010301-DQB1*060101	protection
(DR11) DRB1*110101-DQA1*050501-DQB1*030101	protection
(DR12) DRB1*120201-DQA1*060101-DQB1*030101	protection
(DR15) DRB1*150101-DQA1*010201-DQB1*060101	protection
(DR16) DRB1*160201-DQA1*010202-DQB1*050201	protection
Except above-mentioned haplotypes	others

ST. 2. Genotype risk groups based on haplotype properties of patients.

Genotype risk group	Haplotype properties
High-risk	susceptibility/ susceptibility
Non-high-risk	susceptibility/ protection
	susceptibility/ others
	protection/ protection
	protection/ others
